# Hip Surveillance and Management of Hip Displacement in Children with Cerebral Palsy: Clinical and Ethical Dilemmas

**DOI:** 10.3390/jcm12041651

**Published:** 2023-02-19

**Authors:** Jason J. Howard, Kate Willoughby, Pam Thomason, Benjamin J. Shore, Kerr Graham, Erich Rutz

**Affiliations:** 1 Nemours Children’s Hospital, Wilmington, DE 19803, USA; 2 Department of Orthopaedics, The Royal Children’s Hospital, Parkville 3052, Australia; 3 The Hugh Williamson Gait Laboratory, The Royal Children’s Hospital, Parkville 3052, Australia; 4 Boston Children’s Hospital, Boston, MA 02115, USA

**Keywords:** cerebral palsy, hip displacement, hip surveillance, GMFCS, adductor-psoas release, hip reconstruction, guided growth, salvage surgery

## Abstract

Hip displacement is the second most common musculoskeletal deformity in children with cerebral palsy. Hip surveillance programs have been implemented in many countries to detect hip displacement early when it is usually asymptomatic. The aim of hip surveillance is to monitor hip development to offer management options to slow or reverse hip displacement, and to provide the best opportunity for good hip health at skeletal maturity. The long-term goal is to avoid the sequelae of late hip dislocation which may include pain, fixed deformity, loss of function and impaired quality of life. The focus of this review is on areas of disagreement, areas where evidence is lacking, ethical dilemmas and areas for future research. There is already broad agreement on how to conduct hip surveillance, using a combination of standardised physical examination measures and radiographic examination of the hips. The frequency is dictated by the risk of hip displacement according to the child’s ambulatory status. Management of both early and late hip displacement is more controversial and the evidence base in key areas is relatively weak. In this review, we summarise the recent literature on hip surveillance and highlight the management dilemmas and controversies. Better understanding of the causes of hip displacement may lead to interventions which target the pathophysiology of hip displacement and the pathological anatomy of the hip in children with cerebral palsy. We have identified the need for more effective and integrated management from early childhood to skeletal maturity. Areas for future research are highlighted and a range of ethical and management dilemmas are discussed.

## 1. Introduction

**Figure 1:**
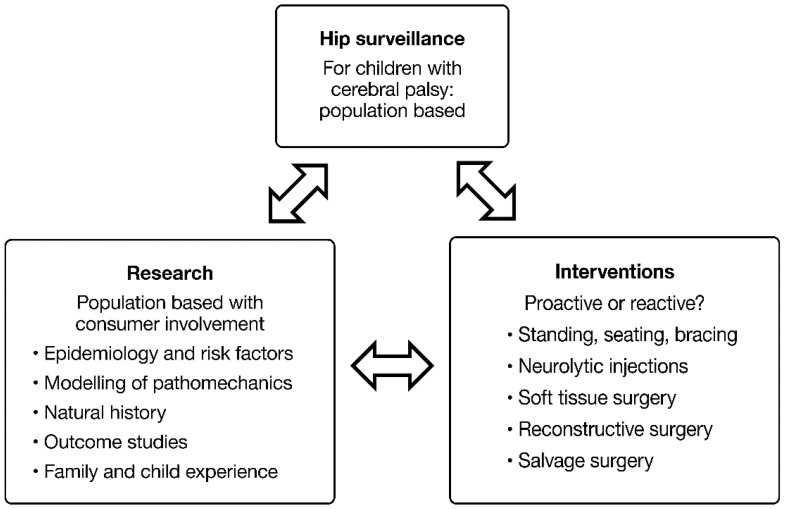
Hip surveillance, research and interventions. Copyright Bill Reid, Pam Thomason and Kerr Graham, RCH Melbourne. Hip surveillance for children with CP improves the knowledge and research base. It can help us understand why hips dislocate and effects of intervention on the natural history of hip migration. Hip surveillance usually leads to interventions, which can be proactive or reactive and include both non-operative and operative approaches. Hip surveillance, research and outcome studies are inextricably linked. Each informs the other.

Hip displacement (HD) is common in non-ambulatory children with cerebral palsy (CP) and may result in pain, limitations in sitting ability and impaired Health Related Quality of Life (HRQoL) [1,2]. In a large, population-based study, the prevalence of HD, defined as migration percentage (MP) > 30%, was 35% for the whole CP population, rising to 90% for children at Gross Motor Function Classification System (GMFCS) Level V [3]. Prevalence was related in a linear fashion to GMFCS Level but not to motor type [1,3]. In recent years, there has been an increasing interest in hip surveillance (HS) and preventive and reconstructive surgery [4,5]. Reports from single-site studies, state-wide studies, national studies and systematic reviews are largely positive about the benefits of HS in detecting HD in CP [5,6,7,8,9,10]. However, questions remain pertaining to the HS process, the efficacy of non-operative treatments, the efficacy of soft tissue releases in younger children, the timing and dose of reconstructive osteotomies and the need for salvage procedures (i.e., joint resection). The common association of multiple co-morbidities, leading to a higher risk of post-operative medical complications, further impacts clinical decision-making [1]. The frailty of some children with severe CP raises clinical and ethical questions about surgical management. The purpose of this review is to identify areas where knowledge is lacking and where future research efforts might be focused to help answer these questions (Figure 1).

## 2. Hip Surveillance for Children with Cerebral Palsy

Detecting a disease early—when it may be easier to treat—is intuitively attractive, but the benefits and potential harms require careful evaluation. Screening and surveillance programs can be successful, but there may be unintended consequences. It has been stated that: ‘all screening programs do harm…some do good as well’ [1,11,12].

**Figure 2:**
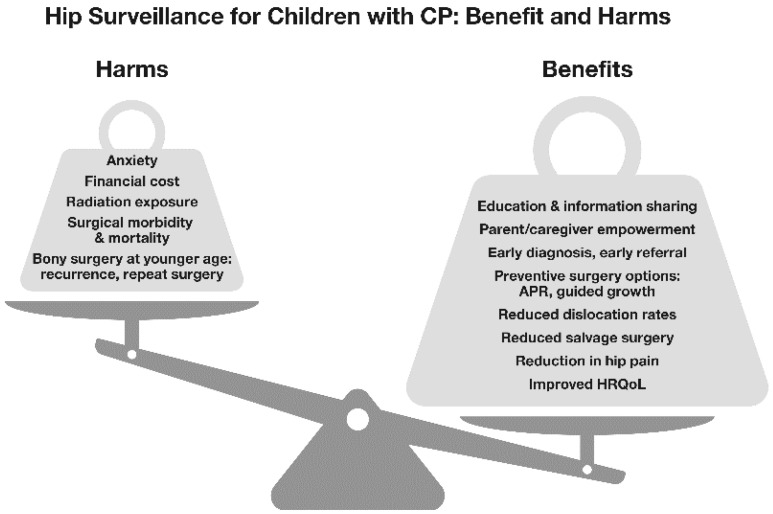
The benefits and harms of hip surveillance. Copyright Bill Reid, Kate Willoughby and Kerr Graham, RCH Melbourne. Some of the potential benefits and harms of hip surveillance are shown as a ‘see-saw’ or ‘teeter-totter’. Through hip surveillance, early identification of hip displacement and referral to orthopaedics provide options for surgical management. This, in turn, introduces additional potential harms and benefits. There is strong evidence that the benefits outweigh the harms for children with cerebral palsy as a population. The benefit to harm experience however is not just for study cohorts or populations, but is experienced by individual children and their families. While early bony surgery is safe and effective in the short term, the associated harm is a high recurrence and surgery revision rate. This could be reduced, perhaps, through ‘temporising’ surgical options such as adductor-psoas muscle release (APR) and/or guided growth. As noted elsewhere, surgery is safer and more successful when conducted in the context of an experienced, multidisciplinary team which includes high volume, experienced surgeons.

HS for children with CP involves both clinical and radiographic examinations based on a risk assessment linked to gross motor function, rather than the development of symptoms, to help subvert a natural history typified by painful osteoarthritis and impairment [1,5,6,7,8,9,10,11]. This may prevent some children from developing symptomatic dislocation if HS detects HD early enough for treatment to make a difference in outcome (Figure 2). Although the reported prevalence of pain in children with CP and dislocated hips is variable, it reached >80% in some studies [13,14]. However, given that some with HD may never develop pain, children may be subjected to physical, emotional and financial harms from interventions that may have little clinical impact [11]. As with many screening programs, the trade-offs between potential benefits and harms relating to HS is complex and not fully understood [11,12] (Figure 2).

Clinicians who have embraced hip surveillance may have been influenced historically by the miseries of neglected hip dislocation in teenagers and young adults with severe CP. On top of marked restrictions in gross motor function and the inability to walk, the development of HD can be a source of pain, impair the ability to sit comfortably and significantly reduce quality of life [2,6].

## 3. Lessons from the History of Hip Surveillance

The first studies on the aetiology and prevention of hip dislocations in children with CP were published more than 70 years ago. Dr. Mercer Rang was one of the first clinicians to advocate for hip surveillance for children with CP and to develop educational materials to promote the concept. He educated clinicians and parents about HD, advocating for yearly clinical and radiographic examinations for children with CP and severe limitations in walking [15]. This remains the core of contemporary hip surveillance [6,7,8,9,10].

Dr Rang produced instructive posters to remind all staff of their responsibility to check children for silent hip dysplasia on a regular basis. He called this approach ‘The Art of Preventive Orthopaedics’. He wryly observed that ‘he had not met a child with CP who had been helped by having a dislocated hip’ [15]. In 1985, he published a study of adductor release surgery in children with CP [16]. This is one of the many studies on the treatment of HD prior to the introduction and widespread use of the GMFCS, a valid and reliable classification of functional severity [1,17]. This makes the data harder to interpret by contemporary standards, given the less reliable classification used at the time emphasizing motor type and topographical patterns [1,18]. At a mean follow-up of approximately 4 years, Dr Rang and his colleagues reported good outcomes in 80% of children and identified predictive factors for success or failure following adductor surgery [16]. Implicit in Dr. Rang’s publications was the principle that dislocation could be prevented in the majority of children with CP by a single, well-timed soft tissue surgery, adductor-psoas release (APR). Unfortunately, a large recent population-based study incorporating the GMFCS does not support this optimistic view of APR surgery, which in turn changes the benefit-to-harm calculation with respect to the application of HS to determine the need and timing of surgery for HD [19] (Figure 2).

HS programs were introduced and refined in Southern Sweden (CPUP) and in Australia over the past 20–30 years [6,7,8,9]. HS has also been endorsed and supported by numerous professional bodies including the Australian Academy of Cerebral Palsy and Developmental Medicine (AusACPDM), the American Academy of Cerebral Palsy and Developmental Medicine (AACPDM) and the Paediatric Orthopaedic Society of North America (POSNA) [7,9,20]. HS programs have been introduced in many other countries as well, including the United Kingdom, Canada and India [5,19,21,22].

Children with developmental dysplasia of the hip (DDH) are screened most intensively in the neonatal period because the majority of hips are unstable at birth. Children with CP usually have normal hips at birth, and surveillance rather than screening is applied for children who are considered to be at high risk of HD, especially those who are non-ambulant (GMFCS IV and V) [3,23]. Clinical examination—involving the assessment of hip adductor and flexor contractures—is important in the overall assessment of the child, but it is unreliable in detecting hip displacement and incomplete without radiology [15,23]. HS programs are based on regular high-quality radiographs of the hips with the timing and frequency determined by studies which have identified the relative risk according to GMFCS level [3,23]. The extent that the femoral head is uncovered by the acetabular roof is measured from pelvic radiographs using the migration percentage (MP), a quantitative measure of HD with face validity and acceptable reliability [20,22,23,24,25]. The predictors and associations of HD in CP have been studied and reported but there is no universally agreed threshold for MP to define significant HD, to indicate referral to orthopaedic surgeons, or to determine the need for preventive, reconstructive or salvage surgery [24,25,26].

**Figure 3:**
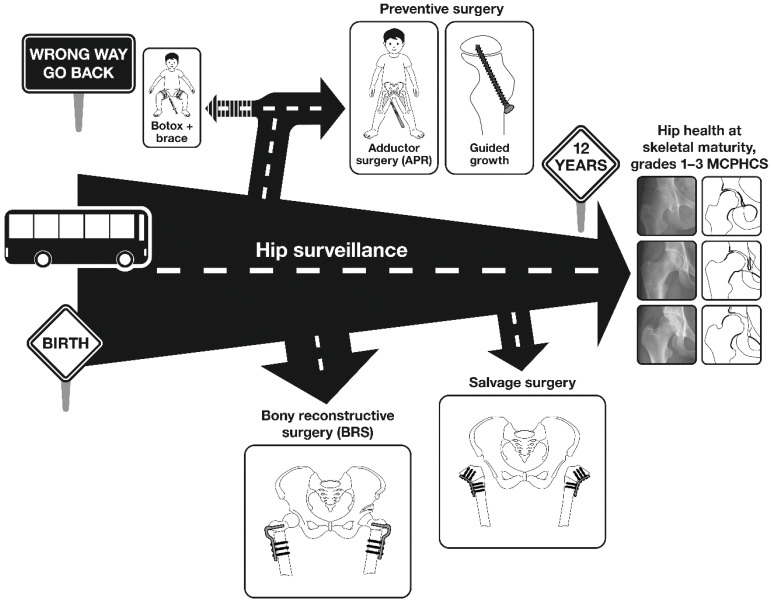
The hip surveillance journey. Copyright Bill Reid, Pam Thomason and Kerr Graham, RCH Melbourne.

Within a MP threshold range of 30–40%, referral to an orthopaedic service is usually advised [20,21,22,23]. Unlike DDH, non-operative treatments for cerebral palsy hip displacement (CPHD) are relatively ineffective [27]. That said, operative treatments are invasive and have substantial failure rates and risks of medical and surgical complications [1,28,29]. The preventive APR surgery, initially advocated by Dr. Rang, has a high failure rate in recent, longer-term studies [16,19,30,31,32]. It is now apparent that a substantial percentage of children with severe CP (GMFCS IV and V) will require bony reconstructive surgery to maintain good ‘hip health’ [32]. Some may require revision or repeated episodes of bony reconstructive surgery to achieve acceptable hip morphology at skeletal maturity [30,31,32] (Figure 3). This knowledge contributes to the ethical and clinical dilemmas surrounding HS and the management of asymptomatic HD in young people with CP. How many operations are acceptable to children, parents and surgeons for a condition which, at the time of first diagnosis, is often asymptomatic [1,3]?

Figure 3 illustrates the journey of a child with CP, from birth towards skeletal maturity as a road travelled with various exits and destinations. There is an interaction between hip surveillance and options for preventive and reconstructive surgery, with the goal of avoiding salvage surgery and reaching skeletal maturity with good hip health, defined in broad terms a pain-free, mobile hip with morphology at Melbourne Cerebral Palsy Hip Classification Scale (MCPHCS) Grades 1–3 (Supplementary Materials S1).

At birth, the hip is normal. In early childhood, hip displacement is usually silent and the rate of increase in MP can be between 6% and 12% per annum, for children functioning at GMFCS V. 

The option for children of BoNT-A and bracing of the hip has been shown to be ineffective: ‘wrong way, go back!’

The second preventive option is adductor psoas release (APR) surgery. In some children, APR surgery can stop the progression of MP and in others the rate of progression is reduced. After APR surgery, the number of hips which will stabilise for the long-term is not clearly known. APR surgery has been used for approximately 80 years and the results in the literature are mixed. In contrast, the third preventive option, ‘guided growth’ is a minimally invasive, relatively new intervention which may have a major impact on the femoral deformity, in younger children who are growing rapidly. Studies to date are short-term and many questions remain unanswered. This technology requires both long-term studies and controlled studies to define its role.

As indicated in the figure, hip surveillance should continue and if there is a continued progression in MP after APR or guided growth, the next exit is for bony reconstructive surgery. This is most often between the ages of 6 and 12 years, before irreversible femoral head damage occurs. Severe femoral head damage may leave salvage surgery as the only option. The road in the diagram is shown to narrow, indicating that in the second decade the hip and the child may run out of time and out of options. By the time of the adolescent growth spurt, some hips will have dislocated and have irreversible damage leaving salvage surgery as the only option for the hip, the child and the family.

## 4. How Do We Judge the Success of Hip Surveillance?

The goal of HS can be defined narrowly as: the detection of significant HD in children with CP within a given population. This can facilitate timely referral to an orthopaedic surgeon and the option for early intervention. Using such criteria, Kentish and colleagues demonstrated that this goal could be achieved in a large state with low population density and maintained over a 5-year period, despite the many geographical and health care delivery challenges [7]. However, it may not be logical to separate the process of HS from the outcome of management of HD. The purpose of HS is not referral alone, but instead is to facilitate the maintenance of ‘hip health’ in the most vulnerable children with CP [13]. Good ‘hip health’ describes a hip which is pain-free and mobile, without significant fixed deformity. Good ‘hip health’ facilitates comfortable sitting, standing and walking in some children. It also facilitates activities of daily living (ADLs) such as dressing, bathing and perineal hygiene. The hip morphology required to meet these demands varies by GMFCS level and has not yet been precisely defined. Hips with MP < 40% and which are Grades 1–3 according to the Melbourne Cerebral Palsy Hip Classification Scale (MCPHCS E&R, Supplementary Materials S1) have a combination of good or acceptable hip morphology and low pain scores. These children and youth are typically able to sit comfortably with few restrictions [13,33] (Figure 3).

There are several population-based studies which show that HS improves the outcome of CPHD, favouring preventive and reconstructive procedures over salvage surgery [6,8,34]. Prior to HS, children with CP in one tertiary centre were presenting for salvage surgery at a rate of 4–8 per annum. After the introduction of HS, the rates for preventive and reconstructive surgeries increased while salvage surgery was temporarily eliminated [6]. In Southern Sweden, the 20-year results of hip surveillance, combined with a responsive surgical program, resulted in very low rates of dislocated hips and salvage surgery [8]. A contrary view was expressed by Larsen et al. who reported that HS had failed to reduce hip pain in adolescents at long-term follow-up [35]. Perhaps it was the surgical program that failed to reduce hip pain rather than the HS [35].

## 5. Net Zero Is Neither Possible Nor Desirable

A reasonable goal of HS would be detection of all hips with MP > 30% and referral for assessment and consideration of orthopaedic surgery [6,7,8,9,10]. This should result in a reduction in late dislocations and the need for salvage surgery to its lowest possible level [6,8,32,34]. Achieving a zero rate of late hip dislocation—and the associated salvage surgery—is probably not attainable. Children with advanced hip disease may move from a country or state without HS to one with state-wide HS [8]. The parents of some children with CP are willing to have their children enrolled in a HS program but decline the option of invasive surgery, especially when their children are asymptomatic. A number of children at GMFCS V have limited life expectancy and may be too frail for bony reconstructive surgery [1,36]. There is a danger that frail children detected by HS as having progressive increase in MP might be offered invasive surgery and suffer premature mortality. Every child with CP is a candidate for HS but some may not be candidates for hip surgery [28,29,35] (Figure 2). Approximately 20% of children with severe CP suffer premature mortality by the age of 4 years, without prior surgical intervention [1,36]. Withholding surgical intervention for children with severe CP and HD presents an ethical dilemma, as it is difficult to determine which children would be at higher risk of mortality after hip surgery. The complication rate after hip surgery in non-ambulant children is high and when ‘failure to cure’ is included, the complication rate rose to 54% in one study [28].

## 6. Knowledge Gaps with Respect to Hip Surveillance

Given the high prevalence of CPHD in non-ambulant children, those functioning at GMFCS IV and V have been the focus of research in this area [3]. Although the prevalence is lower in ambulant children, the sequelae of late dislocation are magnified by higher functional demands and communication ability [1,3,17,18]. The majority of children with hemiplegia function at GMFCS level I or II, rarely, if ever, suffer hip dislocation [1,3,18]. Late-onset, symptomatic hip subluxation in the second decade, however, can be a problem in children with Type IV hemiplegia, according to the Winters Gage Hicks Classification (WGH) [37]. These children have flexion, adduction and internal rotation alignment of the hip and may present de novo with hip pain if not under regular HS. Although the prevalence is low, we are not aware of population-based studies which identify the precise rate of HD in Type IV hemiplegia. Smaller studies of surgical outcomes of symptomatic HD in Type IV hemiplegia suggest that this can be a difficult management problem, often requiring major reconstructive surgery [37]. A better understanding of the natural history of HD in hemiplegia would be very useful to guide the practice of HS for these children, the largest subgroup of children with CP [3,38]. Although the Type IV hemiplegia phenotype can be recognised by a combination of clinical examinations and three-dimensional gait analysis, the WGH Types are overlapping, not discrete and there are problems with the reliability of the classification [1,38].

Ambulant children with diplegia (GMFCS I-III) have lower rates of HD but natural history, optimum HS guidelines and clinical management have not been well studied [3]. Miller and colleagues recently reported four children with asymmetric diplegic CP functioning at GMFCS levels II and III, with the more involved hip showing rapid, progressive displacement at a later age [39]. They noted the current HS guidelines may not adequately identify HD in children with asymmetric diplegia and pelvic obliquity. Modifications to current HS guidelines may be warranted [39].

It has been assumed that the risk of progressive HD is negligible after triradiate cartilage (TRC) closure—a proxy for the onset of skeletal maturity—and thus HS could safely be discontinued after that point [9]. A recent large study, however, has demonstrated that there is a risk of further hip displacement in children with CP after closure of the TRC especially for those with risk factors including male gender, pelvic obliquity, high MP and no prior surgery [40]. For those youth at risk, the authors suggested that HS be continued up until age 18 years, with at least an x-ray every 2 years [40].

## 7. Hip Surveillance Leads to Bony Reconstructive Surgery When Children Are Younger

The treatment effects of all interventions are weak in comparison to the biomechanical forces which predispose the hip to primary or recurrent displacement [3]. Most operative interventions lead to an improvement in MP or a reduced rate of progression in MP [16,19,30,31,32] (Figure 3). Despite the release of adductors, flexors and proximal hamstrings, there is a tendency to progressive HD and, if HS is neglected, dislocation [19,28,32,33]. Recent thinking points to abnormalities in proximal femoral growth secondary to a lack of functional ambulation, rather than muscle imbalance due to spastic contractures alone, as being causative for CPHD [41]. Management must include strategies which ensure stable, pain-free hips with good morphology, after skeletal maturity [13]. Outcome studies with follow-up to an age younger than skeletal maturity are provisional, at best (Figure 3).

Early detection of HD by HS leads to early referral to orthopaedic surgery. When APR is the first-choice preventive option, early failure will often lead to consideration of bony reconstructive surgery (BRS) at an earlier age [13,19] (Figure 3). This might seem to be a good thing, with early bony correction of proximal femur and acetabular deformities intuitively leading to better outcomes. However, the success of bony reconstructive surgery is inversely related to the age at surgery [28,30,32]. Younger children have more years of growth ahead of them during which re-displacement may occur [33,40]. This is logical when it is remembered that the factors which contribute to hip displacement—such as adductor spasticity, weakness of the hip abductors and limited weight-bearing—remain present and largely unchanged after orthopaedic surgery [1,3,17]. The risk period for recurrence is mainly, but not exclusively, related to the interval between bony reconstructive surgery and skeletal maturity [40].

## 8. The Cause of Hip Displacement in Children with Cerebral Palsy: Spasticity or Weakness?

**Figure 4:**
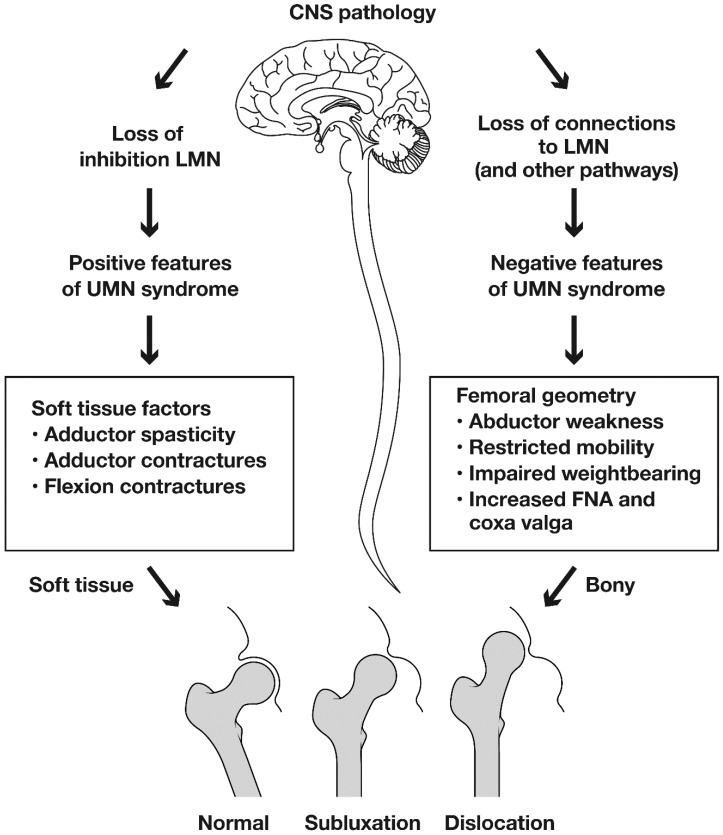
The pathophysiology of hip displacement in children with cerebral palsy. Copyright Bill Reid, Jason Howard and Kerr Graham, RCH Melbourne.

Hip displacement and dislocation have historically been linked to muscle imbalance across the hip joint and spasticity in the hip adductors and flexors [15,16,42]. There is evidence from musculoskeletal modelling to support the role of muscle imbalance and increased contact forces [42]. However, modelling depends on forces measured during gait, and extrapolation of these data to the non-ambulant child are problematic. In recent years the emphasis has shifted to include the role of abnormal proximal femoral geometry. In population-based studies of CPHD, the prevalence of displacement (MP > 30%) was directly related to GMFCS level, but not to motor type [3]. Mean femoral neck anteversion (FNA) and neck shaft angle (NSA) increased stepwise from GMFCS I to V [43,44]. In children with hypotonia, the prevalence of CPHD was the same as for children with hypertonia [3,41]. These data suggest that activity limitations may make a stronger contribution to HD than adductor spasticity [1,3,17,41,42,43]. A fundamental question is whether abnormal femoral geometry is a primary response to activity limitations and hip abductor weakness, or a secondary response to spastic contractures of the hip adductors and flexors [3,39,42]. Ulusaloglu and colleagues compared proximal femoral and acetabular geometry in non-ambulatory children with spastic CP (GMFCS IV, V) to non-ambulatory children with spinal muscular atrophy (SMA, Types I and II). They reported an earlier onset of HD in SMA, with similar radiographic features in the two groups, despite CP being characterised by spasticity and SMA by hypotonia [41]. A recent study identified laxity of the capsule and ligaments as the primary pathology in HD in children with CP, but offered no supporting evidence [45]. Whilst joint laxity is a fundamental factor in DDH there is no clinical or experimental evidence to suggest that it plays a primary role in CPHD [3,39,41,42,45]. Understanding pathological anatomy and pathophysiology of CPHD is important as it may have a major role in the design, planning and selection of management strategies (Figure 3 and Figure 4).

The cause of hip displacement in children with severe CP has been considered to be secondary to the positive features of the UMN Syndrome, which include adductor spasticity and contractures of the hip adductors and flexors. This leads to muscle imbalance across the hip joint. More recently, the negative features of the UMN Syndrome have also been considered and these include profound alterations in proximal femoral geometry. It is hypothesised that abductor weakness and limitations in weight-bearing lead to persistence of femoral neck anteversion (FNA), progressive lateral tilting of the proximal femoral epiphysis leading to increasing head shaft angle (HSA) and coxa valga or increased neck shaft angle (NSA). Probably it is a combination of both the positive and negative features of the UMN Syndrome which have such a strong deleterious effect on the growing hip in children with severe CP. Guided growth may be a logical intervention to directly address abnormal proximal femoral geometry.

The emphasis on adductor spasticity and contracture has led to an emphasis on abduction bracing, spasticity reduction by injections of Botulinum Neurotoxin A (BoNT-A) and APR surgery [45,46,47]. The outcomes of these interventions, in comparison to bony reconstructive surgery which addresses the abnormal proximal femoral geometry, are unpredictable and often disappointing [48]. The identification of persistent or recurrent coxa valga and secondary acetabular dysplasia in non-ambulant children with CP, suggests a possible role for guided growth of the proximal femur [49,50]. We suggest that muscle imbalance *and* abnormal proximal femoral geometry acting together may contribute to CPHD which we suggest is a more accurate term than ‘spastic hip disease’ [1,3,41,44].

## 9. The Natural History of Hip Displacement in Children with Cerebral Palsy

There are few studies of the natural history of hip displacement in children with CP since children who are receiving HS are usually offered early intervention, while those not receiving HS tend to present late, often with an established, painful dislocation [51]. What is known is that most hips in children with CP are normal at birth, although a very small number have congenital dislocations, such as in DDH [51]. In a large study of untreated children with CP, dislocation occurred in 10% of children by progressive lateral displacement of the femoral head from the acetabulum [51]. This resulted in secondary acetabular dysplasia, femoral head deformity and frank arthrosis [6]. The prevalence of painful degenerative arthritis in this cohort was very high, with the majority presenting around the time of the pubertal growth spurt requiring salvage surgery [6]. More recently, Terjersen reported a cohort of 76 children with CP who were followed for five years before treatment [52]. Age and ambulatory ability were the main predictors of the speed of HD. Children who could not walk had a 12% increase in MP per annum compared to 2% in children who could walk, with or without support [52]. In CPHD, abductor weakness and ambulatory ability may be more important than adductor spasticity [1,43].

For children functioning at GMFCS Level V, the annual increase in MP in one study was 6.2%. The annual increase in femoral neck shaft angle was 3.4 degrees. This confirms the contributions of progressive coxa valga to lateral displacement of the femoral head from the acetabulum and secondary acetabular dysplasia. [53]. Fortunately, in a comprehensive review, Lins and colleagues have demonstrated that this bleak natural history can be favourably improved by appropriate HS and timely intervention [54].

## 10. Non-Operative Treatment for Early CPHD

Specialised seating and orthotic systems have been trialled for children with severe CP with varying outcomes with respect to managing HD. The majority of studies were uncontrolled and had short-term follow-up [25]. There is limited evidence that specialised seating and orthotic devices can prevent progressive HD in non-ambulant children with CP [25]. Given the strong natural history leading to hip dislocation, it would be surprising if these forces could be negated by an orthotic device. It would also seem improbable, from first principles, that the progressive coxa valga seen in non-ambulatory children with CP could be reversed by non-operative means [43,44,45].

Despite this, a recent study of a novel hip brace reported slowing of HD by daily wear of at least 12 h [45]. Unfortunately, participants were not matched in terms of baseline MP. The authors suggest that a primary aetiological factor in CPHD is laxity of the joint capsule and surrounding ligaments. It was claimed that the brace was designed to reduce coxa valga but no evidence was offered in support of this improbable claim [45]. Given the failure of other non-operative modalities over the long-term, despite short-term success, longitudinal follow-up of this cohort is awaited with interest [13,45,46].

## 11. Injections of Botulinum Neurotoxin A Combined with Bracing

Injection of botulinum toxin A (BoNT-A) to the hip adductors, hip flexors and hamstrings has been practiced for many years for the reduction of focal spasticity in children with CP [46,47]. However, the evidence that repeated injections of BoNT-A can prevent or even slow HD is mixed. Repeated injections of BoNT-A combined with an abduction brace did not prevent progressive HD in a large, randomised clinical trial of three years duration [46]. There was a delay in progression to surgery but no difference in long-term outcomes. The outcomes reported were: the amount of surgery required, final hip morphology and pain levels [13]. There are several short-term studies which reported more favourable outcomes but without a control group and long-term follow-up, so the evidence is not compelling [47]. At the population level, injections of BoNT-A had no impact on the prevalence of CPHD or scoliosis [55].

## 12. Adductor-Psoas Releases for CPHD: 80% Success or 80% Failure?

Both Silver et al. and Presedo et al. reported high success rates from early adductor surgery in non-ambulant children with CP [16,56]. However, these early studies pre-date the use of the GMFCS to stratify patient risk, and both studies had relatively short-term follow-up. With a combination of risk stratification by GMFCS and long-term follow-up, the 60–80% success rate of soft tissue surgery is replaced by a 60–80% failure rate in children functioning at GMFCS IV and V [17,28]. This led Shore and colleagues to the conclusion that ‘adductor surgery works least for those children who need it most’ [17].

**Figure 5:**
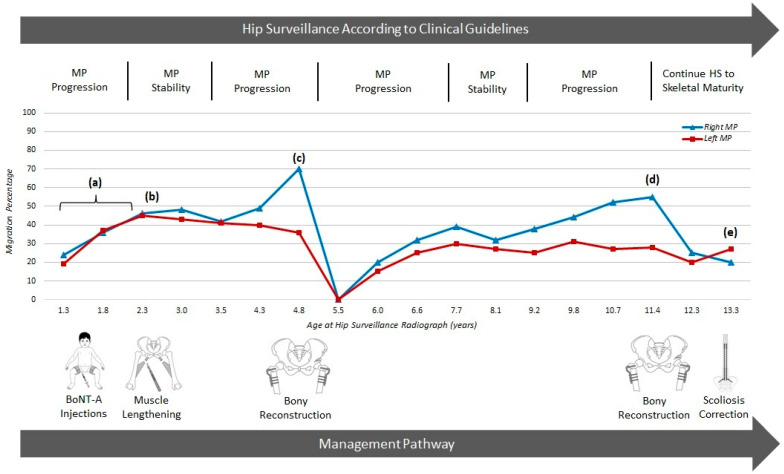
Hip surveillance and response to interventions for hip displacement for a child from age 1–13 years. Copyright Kate Willoughby, RCH Melbourne. The red line shows serial MP for the left hip; the blue line shows serial MP for the right hip, over time. This figure shows the interplay between the hip surveillance and management pathways of a girl who functioned at GMFCS IV and had regular HS and multiple interventions over a 13-year period.

When hip surveillance leads to the identification of early hip displacement in children with no symptoms, it would be ideal to offer a minimally invasive soft tissue surgical approach with good long-term outcomes [11,15]. Adductor surgery seems to work best for younger children with MP between 30% and 50% and abduction <30 degrees [15,30,31] (Figure 5). For many children, a temporary improvement or decreased progression rate in MP can be anticipated after APR. With long-term follow-up, the improvement in MP may continue in ambulant children but a relapse is common after 3–10 years, with continued progression of hip displacement in non-ambulant children [17].

Hip surveillance commenced according to national guidelines with the first radiograph soon after her first birthday. No benefit was gained from repeated injections of BoNT-A and bracing, with progression in MP recorded (a). The treatment with BoNT-A was during the era when the efficacy of this intervention for hip displacement was undecided. APR surgery at 2 years of age (b) resulted in a stable MP for only 2 years. Rapid progression in MP led to bilateral VDROs at age 4 years (c) and the MPs returned to zero. Guided growth may have been a better option at such a young age. MPs gradually increased over the next 6 years and a second episode of bony reconstructive surgery was performed at age 11 years (d), followed by posterior spinal fusion at age 13 years (e). Approaching skeletal maturity, the patient has a level pelvis, mobile pain-free hips which are MCPHCS Grade 3. This has been achieved at significant cost to the child, family and the health care service. More durable interventions and fewer episodes of major intervention are required.

The contemporary view of APR surgery as a primary intervention for HD is that it is a useful intervention, but more often to delay the need for bony reconstructive surgery than a means to achieve long-term hip stability in non-ambulant children [43] (Figure 3 and Figure 5). More prospective studies are required to see if there are sub-groups of non-ambulant children who by virtue of age, low MP or other factors might respond more favourably to APR surgery [11,16,30,31]. Chemo-denervation of the obturator nerve at the time of APR surgery may reduce post-operative pain and spasm, and might prolong the effect on hip stability [43]. However, no comparative studies or clinical trials have been conducted.

## 13. Guided Growth and Proximal Femoral Geometry in CPHD

Given the limited efficacy for APR in non-ambulant children, and the relatively high recurrence rate after reconstructive osteotomies for younger children (<6 years old), the most optimal treatment of early detected CPHD has yet to be determined [28,32,56,57]. To avoid major, invasive bony reconstructive surgery, there has been an increased interest in recent years in using guided growth in the proximal femur to correct some of the bony deformities that develop in non-ambulant children [44]. These include coxa valga, a horizontal tilt to the proximal femoral epiphysis and increased neck-shaft-angle (NSA). Increased head-shaft angle (HSA) is another useful radiological index to describe the abnormal proximal femoral geometry in children with CP, defined as the angle between the proximal femoral growth plate and the femoral shaft [58]. In typically developing children, HSA starts high and decreases over time, consistent with the acquisition of weight-bearing and ambulation [59]. Concurrently, femoral NSA also decreases, likely related to this medialisation of the proximal femoral physis. By contrast, in children with CP, the HSA starts high and remains high, associated with increased coxa valga, progressive increase in MP and secondary acetabular dysplasia [41,59]. These changes have been theorised to be linked to abnormal joint forces and result in these changes in proximal femoral growth, and thus geometry [41,44,59].

If abnormal growth is the primary driver, then interventions which modulate the growth of the proximal femur—by reversal of coxa valga—would seem logical. In addition, guided growth has the potential to provide dynamic correction of proximal femoral deformity during growth in contrast to the acute but static correction offered by hip reconstruction [43,48]. Hsieh and colleagues reported the use of inferomedial screw epiphysiodesis of the proximal femur to reduce the lateral physeal tilt, in the proximal femoral physis [60]. In their study, they reported significant decreases in HSA, MP and AI following guided growth, with longer duration of follow-up and smaller index MP having better outcomes. Further, in their comparative study of adductor surgery, Sheu and colleagues reported significant reductions in MP and a lower rate of MP > 40% in the group augmented with proximal femoral guided growth [61]. The reduction in MP, though significant, was relatively modest in this study, perhaps related to the older age at index procedure (mean 8.1 years). A recent systematic review investigating the role of guided growth in children with CP (178 hips) also reported significant reductions in MP (35% to 26%), HSA (162° to 149°) and AI (22° to 18°), but the mean age at surgery was quite high at 7.2 years. The studies also included many ambulant children who are known to be at low risk of progressive hip displacement, and the mean follow-up was too short for strong recommendations [40,50].

**Figure 6:**
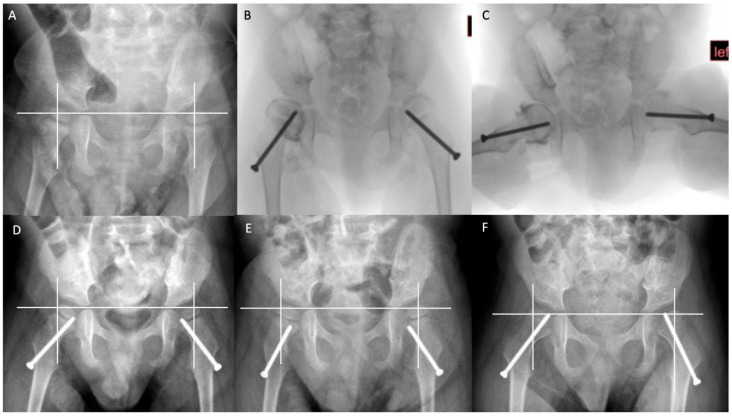
Guided growth in the management of the younger child with early, severe hip displacement. Copyright Jason J Howard, Nemours Children’s Hospital. Serial radiographs of a 3-year-old boy with severe cerebral palsy, functioning at GMFCS V, with progressive hip displacement, left greater than right. (**A**) Pre-operative AP radiograph, MP: right 34%, left 67%. (**B**,**C**) Intra-operative fluoroscopic images showing bilateral hip arthrograms and inferomedial proximal femoral screw epiphysiodesis also known as ‘guided growth’. (**D**) Five-month post-operative follow-up radiograph. (**E**) Twelve-month post-operative follow-up radiograph. Note that the epiphysis has ‘grown off’ the screw bilaterally due to high growth velocity at this early age. (**F**) Sixteen-month post-operative follow-up radiograph, post screw exchange. MP: right 25%, left 27%. Images courtesy Jason J. Howard.

Given these promising outcomes, coupled with limited efficacy for APR alone for non-ambulant children with early HD, two of our authors’ (JJH, BJS) institutions have maintained typical indications for APR surgery (MP > 40% or MP > 30% and progressive) but have augmented this treatment with the use of guided growth. This is achieved with the percutaneous placement of a cannulated screw across the inferomedial proximal femoral physis (Figure 6). For children under 5 years of age, substantial improvements in MP have been realised with this additional treatment, along with a low complication rate. Future comparative studies are planned to substantiate these favourable early results and to determine the longer-term outcomes of this minimally invasive treatment. Whether it will offer a convincing role as an index surgery for hip displacement remains to be seen. Inserting a screw across the proximal femoral physis every 12 to 24 months carries some risks of general anaesthesia, but much less than what would be expected from early reconstructive osteotomies [50].

## 14. Bony Reconstructive Surgery for CPHD

At present, the gold standard intervention for the prevention of progressive hip displacement and for the correction of hip dislocation is a one-stage hip reconstruction [43,48]. This consists of a bilateral adductor lengthening, bilateral varus derotation osteotomies of the proximal femur (VDRO) and pelvic osteotomy or acetabuloplasty, when indicated. There are many cohort studies which report a high success rate in terms of improved hip morphology at medium- to long-term follow-up. However, many studies report high rates of complications including mortality [28,29,62,63,64,65,66]. In one study, the mortality rate was 4% and the overall complication rate was 25%, rising to 68% in children with gastrostomies or tracheostomies [62]. These rates may not be acceptable to the parents of children with CP who have asymptomatic hip dysplasia detected by routine HS.

Recurrent hip displacement and avascular necrosis are two of the most disappointing late outcomes after reconstructive surgery [28,62,63,64,65,66]. Earlier studies of AVN after hip reconstruction in children with CP used or adapted the classifications for AVN in DDH [64,65]. In contrast, Park and colleagues devised a new classification and reported a prevalence of 43% [66]. They reported both risk factors and outcomes.

Despite the significant morbidity and mortality which is associated with bony reconstructive surgery, this surgery has been reported to have good short-term and long-term outcomes [43,48]. Bony reconstructive surgery may be preferable to the uncertain results of salvage hip surgery [1,6,43]. For parents and carers to make an informed choice, the treating team must be aware of their teams’ success rate, failure rate and complication rate [48,57].

The components of a full hip reconstruction are typically adductor lengthening, VDRO and acetabuloplasty. The indications for open reduction and capsulorraphy are not well-defined and there is little published evidence to guide decision-making. Rates for capsulotomy and capsulorraphy vary in the literature and may be related to the utilisation of hip surveillance and the severity and duration of hip dislocation for the specific patient and for the population of children with CP [43,48,57]. Dynamic arthrography is a promising tool to guide intra-operative decision-making as to when acetabuloplasty may be required in BRS, after VDRO. Arthrographic assessment of the labrum after VDRO provides objective information to help surgeons standardise decision-making regarding the indications for acetabuloplasty [43].

## 15. Bilateral Surgery for Unilateral Hip Displacement?

**Figure 7:**
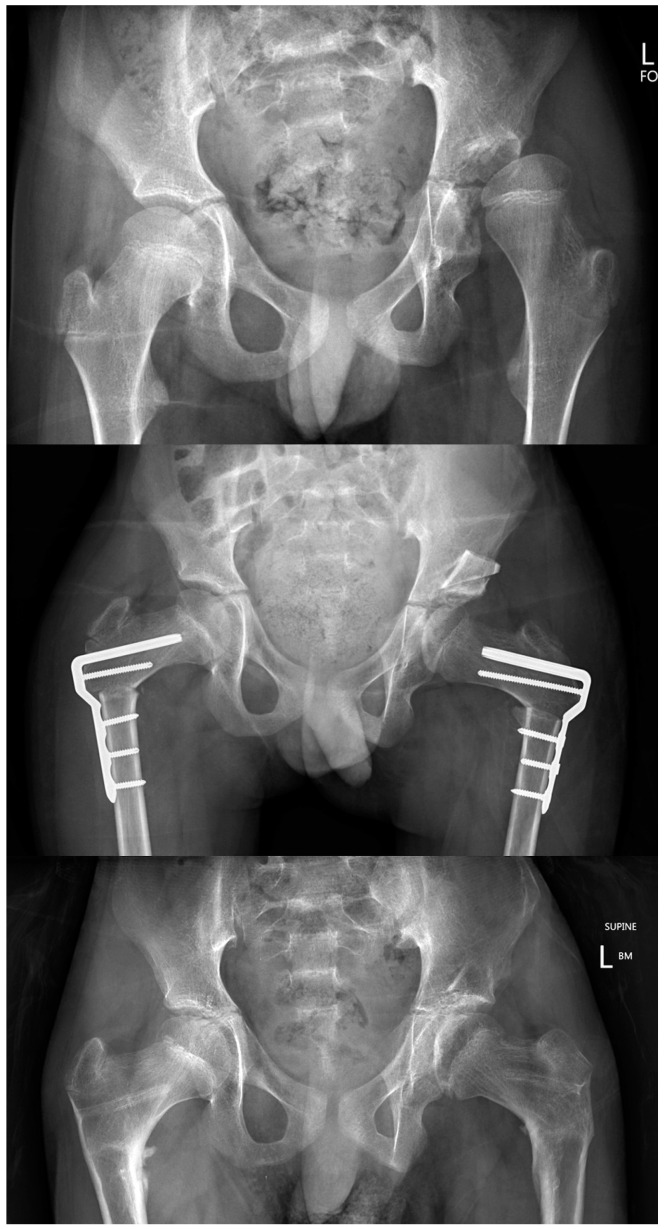
Bilateral surgery for unilateral CPHD. Copyright Kerr Graham, RCH Melbourne. These are the radiographs of a boy aged 10 years, GMFCS IV with clinical and radiographic unilateral (left) CPHD. The top image is immediately pre-operative. The middle image is 7 days after bilateral APR, bilateral VDRO and left San Diego Acetabuloplasty. The bottom image is at 2-year follow-up, after removal of implants. Bilateral surgery gives the best opportunity for symmetry and durability of outcome with marginal increases in surgical adverse events and morbidity.

In children who are functioning at GMFCS IV and V HD may, on MP alone, appear to be unilateral or asymmetric (Figure 7). Some children may present with MP > 40% in only one hip but both hips have similar degrees of increased femoral neck anteversion and coxa valga. Increased MP may be related to the severity of adductor spasticity and contracture, but abnormalities in proximal femoral geometry seem to be more related to the biomechanical environment of the growing hip. Specific factors include GMFCS level, hip abductor weakness and limitations in weight bearing [3,17,44]. Unilateral APR or bony reconstructive surgery are associated with a high rate of displacement in the contralateral hip in long-term follow-up [1,43]. Most surgeons recommend bilateral APR and bilateral reconstructive surgery [43,48,67,68,69]. Bilateral surgery is more durable, maintains symmetry and has only marginal increased risks of morbidity [68,69] (Figure 7).

## 16. Reconstructive Surgical Outcomes and Surgeon Volume

An additional clinical and ethical dilemma is posed by the observation by Shore and colleagues that the outcomes of reconstructive surgery in children with CP are influenced by surgeon volume [57]. In a large study from a specialist tertiary centre, surgeon volume was an independent predictor of the need for surgical revision [57]. The importance of surgeon volume has been identified in many areas of surgical practice. The importance of surgeon volume to outcomes requires further study in CPHD. For the present, we suggest that when children with CPHD require surgery, it should ideally be by a high-volume surgeon, working in a specialised tertiary centre, well-supported by a multidisciplinary medical, anaesthetic and pain management team [43,57,63].

## 17. Scoliosis and Pelvic Obliquity

The group of children with CP who are most at risk of developing hip displacement/dislocation in the first decade of life are also the children who are most at risk of developing scoliosis in the second decade of life [70,71]. The careful work done through hip surveillance and by the hip reconstruction surgeon can be undone by the sudden development of scoliosis and pelvic obliquity [70,71,72,73,74]. Surgery to correct scoliosis usually involves instrumentation and arthrodesis of the spine from T2 to the pelvis [70]. Hips which have previously been managed by ‘successful’ reconstructive surgery can become symptomatic all over again, following loss of motion in spinal segments after surgery for scoliosis [72]. Despite this, a recent study has reported that scoliosis surgery did not influence a change in MP postoperatively—neither positively nor negatively—regardless of the extent of pelvic obliquity correction [75]. The ideal alignment of the spine–pelvic–femoral axis requires further elucidation [74].

## 18. Femoral Head Deformity: Salvage or Reconstruction?

With respect to bony reconstructive surgery, one of the unknowns is the degree of femoral head deformity which is amendable to reconstruction versus progressing to salvage surgery. Prior to the long-term outcome study by Rutz and colleagues, many surgeons lacked confidence in the ability of the femoral head to remodel following reconstructive surgery [45]. Probably, too many femoral heads have been excised and this may have led to too many children being subjected to salvage surgery [40,43].

A recent study of non-ambulant children with CP investigated the status of femoral head deformity at skeletal maturity, finding an MP > 30% at triradiate cartilage closure was associated with a ‘more severe’ outcome at final follow-up [76]. This more severe head shape was also associated with a higher risk of worse osteoarthritis by Tönnis grading. Interestingly, the authors found that prior reconstructive surgery had no impact on head shape at skeletal maturity which they felt was likely due to a lack of complete containment of the femoral epiphysis. Additional work is required to precisely define the degree of femoral head deformity which is amendable to reconstructive surgery versus the degree of deformity which would be better managed by a salvage surgical option [45,76].

## 19. Palliation of CPHD and the Role of Salvage Surgery

Some clinicians are not convinced by the value of hip surveillance and the need for bony reconstructive surgery in the management of CPHD in the non-ambulant child. This is supported by the observation that not all dislocated hips will be symptomatic and therefore some adopt a reactive approach, meaning to offer intervention only when hips become symptomatic [5]. The only hips in our centre which did not become symptomatic at long-term follow-up were a minority of children with early, complete, bilateral dislocations [6,13]. It might be appropriate to consider a reactive approach for asymptomatic, bilateral dislocations, especially in frail children functioning at GMFCS V with limited life expectancy [36].

The range of surgical procedures described for salvage of a painful dislocated hip in children with CP include at least eight widely used and radically different procedures, from arthrodesis, excision arthroplasty, osteotomy, to total joint replacement [43]. When multiple surgical procedures are offered to treat a single condition, it is often an indication that no one procedure is very effective or acceptable to the target population. The majority of outcome studies from salvage surgery are relatively small case series. There are few comparative studies and follow-up has generally been short- to medium-term. The majority of studies lack adequate documentation of pain before and after surgery as well as valid patient reported outcome measures (PROMS) [43,77,78,79,80]. A notable exception was the study by Koch et al. [79]. They compared the outcomes of three surgical groups to injection of the painful hip with local anaesthetic and corticosteroid. The study was strengthened by a standardised approach to surgery and routine use of visual analogue scales (VAS) to assess pain. The disadvantage of injecting the hip was the need for repeated injections which became less effective over time. Both resection arthroplasty and interposition arthroplasty were effective in reducing pain scores and were supported by parental satisfaction scores [79].

The recent publication of a study which reported racial disparities in both the risk of requiring a salvage procedure and the rate of post-operative complications points to yet another clinical and ethical dilemma in the management of CPHD which, to date, has gone unrecognised and unreported [80]. As this is the first report of this important ethical issue, further studies are required.

## 20. Summary, Conclusions and Future Directions

The purpose of this review is not to undermine confidence in HS, which we think offers the only realistic opportunity to learn about natural history and risk factors for hip displacement in children with CP. This information gives clinicians and parents the ability to have a conversation regarding management options which are not available once a hip dislocates and has reached the ‘point of no return’ in terms of femoral head deformity. However, there are many unanswered questions regarding hip surveillance and the surgical management of hip displacement which require further study.

There is a remarkable level of agreement and congruence in HS guidelines from many countries around the world for children with bilateral, severe CP, GMFCS IV and V. In contrast HS protocols for ambulant children are lacking in strong evidence and this is reflected in uncertainty and variation in HS protocols. This is the key area for future research.

We do not question the need to refer children with hip displacement to orthopaedic surgeons for surgery for the prevention of progressive hip displacement or the reconstruction of displaced hips. It is rather a plea for controlled studies, long-term outcome studies, patient reported outcome measures and family centred studies.

Key areas for future studies include identifying better preventive strategies to avoid BRS, or at least to delay BRS until older age when outcomes are more predictable. The long-term results of APR surgery require further study. The potential role for guided growth as a preventive strategy requires urgent investigation. This would ideally be in the form of comparative surgical studies and clinical trials which include patient-reported outcomes and long-term follow-up.

## Data Availability

Not applicable.

## Supplementary Materials

The following supporting information can be downloaded at: https://www.mdpi.com/article/10.3390/jcm12041651/s1, Supplementary Materials S1: The Melbourne Cerebral Palsy Hip Classification System (MCPHCS) Expanded and Revised.
