# Supplementary material for: Hip Surveillance and Management of Hip Displacement in Children with Cerebral Palsy: Clinical and Ethical Dilemmas
Source: J Clin Med. 2023 Feb 19;12(4):1651. doi: 10.3390/jcm12041651 (PMC9960656; doi:10.3390/jcm12041651)
Supplement: Supplementary file 1 [file jcm-12-01651-s001.zip › jcm-2133575-supplementary material.pdf]

The Melbourne Cerebral Palsy Hip Classification Scale
(Expanded and Revised)

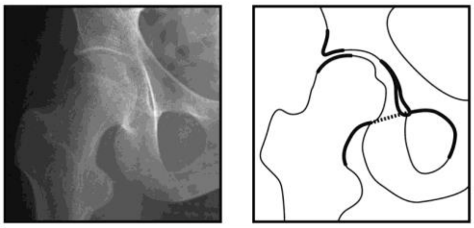

Grade 1: Normal Hip – Migration Percentage <10%

1. Shenton's arch intact
2. Femoral head round (within 2mm using Mose circles)
3. Acetabulum – normal acetabular development with a normal horizontal sourcil, an everted lateral margin and normal tear drop development
4. Pelvic obliquity <5°
5. No degenerative change, no pain

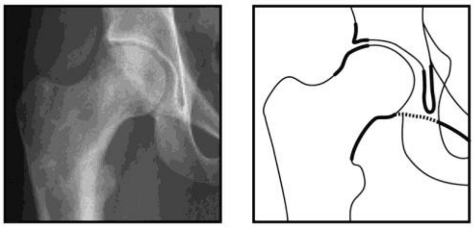

Grade 2: Near Normal Hip – Migration Percentage ≥10% ≤15%

1. Shenton's arch intact
2. Femoral head round or almost round
3. Acetabulum – normal or near normal development
4. Pelvic obliquity <5°
5. Low risk of degenerative change, usually pain free

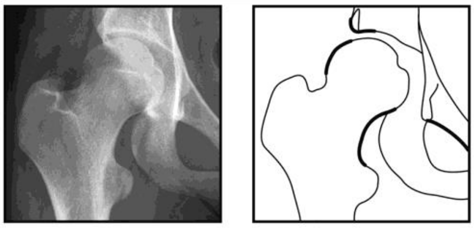

Grade 3: Dysplastic Hip – Migration Percentage >15% ≤30%

1. Shenton's arch intact or broken by ≤5mm
2. Femoral head round or mildly flattened
3. Acetabulum normal or mildly dysplastic including blunting of the acetabular margin and a widened tear drop
4. Pelvic obliquity <10°
5. Low risk of degenerative change, occasionally mild pain

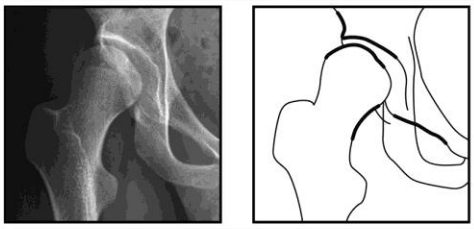

Grade 4: Dysplasia With Mild Subluxation – Migration Percentage >30% <60%

1. Shenton's arch broken by >5mm
2. Femoral head some flattening – Appendix 1
3. Acetabulum dysplastic – Appendix 2
4. Pelvic obliquity variable – Appendix 3
5. Risk of degenerative change, pain variable

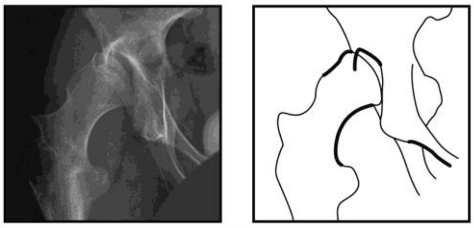

Grade 5: Moderate to Severe Subluxation – Migration Percentage ≥60% <100%

1. Shenton's arch broken by >10mm
2. Femoral head variable deformity – Appendix 1
3. Acetabulum variable deformity – Appendix 2
4. Pelvic obliquity variable – Appendix 3
5. Degenerative change frequent, pain frequent

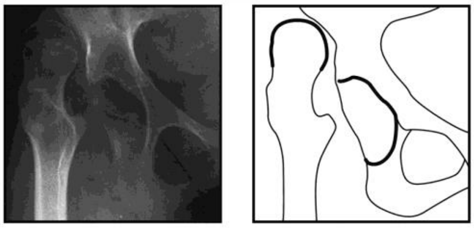

Grade 6: Dislocated Hip – Migration Percentage ≥100%

1. Shenton's arch completely disrupted
2. Femoral head variable deformity – Appendix 1
3. Acetabulum variable deformity – Appendix 2
4. Pelvic obliquity variable – Appendix 3
5. Degenerative change frequent, pain frequent

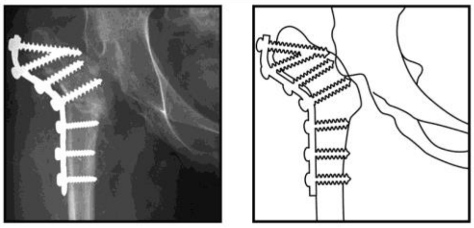

Grade 7: Salvage Surgery

1. Valgus osteotomy
2. Arthrodesis
3. Excision arthroplasty (Castle) ± valgus osteotomy (McHale)
4. Replacement arthroplasty
5. Pain relief following salvage surgery: variable
